# The clinical significance of in-house metagenomic next-generation sequencing for bronchoalveolar lavage fluid diagnostics in patients with lower respiratory tract infections

**DOI:** 10.3389/fcimb.2022.961746

**Published:** 2022-12-15

**Authors:** Shixiao Li, Jiajia Qin, Peng Zhou, Minfei Peng, Jiao Qian, Yingying Cai, Qingxin Shi, Tao-Hsin Tung, Bo Shen, Sufei Yu

**Affiliations:** ^1^ Department of Clinical Microbiology Laboratory, Taizhou Hospital of Zhejiang Province Affiliated to Wenzhou Medical University, Taizhou, Zhejiang, China; ^2^ Department of Pharmacy, Taizhou Hospital of Zhejiang Province Affiliated to Wenzhou Medical University, Taizhou, Zhejiang, China; ^3^ Evidence-Based Medicine Center, Taizhou Hospital of Zhejiang Province Affiliated to Wenzhou Medical University, Taizhou, Zhejiang, China

**Keywords:** metagenomic next-generation sequencing (mNGS), in-house, lower respiratory tract infections, diagnostic, bronchoalveolar lavage fluid

## Abstract

**Objective:**

Metagenomic next-generation sequencing (mNGS) technology has the potential to detect a wide range of pathogenic microorganisms. However, reports on the diagnostic value and clinical significance of different platforms of mNGS for patients with lower respiratory tract infections (LRTIs) remain scarce.

**Methods:**

A total of 306 patients with suspected LRTIs were enrolled from January 2019 to December 2021. The diagnostic performance of conventional methods and mNGS on bronchoalveolar lavage fluid (BALF) were compared. BALF mNGS was performed using a commercial and an in-house laboratory. The diagnostic value and the clinical implications of mNGS for LRTIs were analyzed for the different platforms.

**Results:**

The positive rate of mNGS in the in-house group was higher than that in the commercial group (85.26% *vs*. 70.67%, *p* < 0.001). mNGS significantly increased the pathogen detection rate compared with conventional methods [from 70.67% *vs*. 22.67% (*p* < 0.001) to 85.26% *vs*. 30.77% (*p* < 0.001)]. The pathogens detected using mNGS included bacteria, fungi, viruses, and atypical pathogens. The in-house platform performed well on a wider spectrum of microbial distribution. Furthermore, it showed an advantage in detecting mixed pathogens in immunocompromised patients. Among the mNGS positive cases, 34 (32.0%) cases had their antibiotics adjusted in the commercial group, while 51 (38.3%) cases had a change of treatment in the in-house group. Moreover, the turnaround time of mNGS and the time from mNGS to discharge in the in-house group were significantly shorter than those in the commercial group.

**Conclusion:**

In-house mNGS had a higher detection rate and can show a wider spectrum of pathogens, with potential benefits for the clinic by shortening the turnaround time and hospitalization, and it may be more suitable for clinical microbiology laboratories.

## Introduction

Lower respiratory tract infections (LRTIs) represent one of the most common global health problems with high incidence and mortality rates, especially in the elderly and immunocompromised adults (Langelier et al., 2018; Claassen-Weitz et al., 2021). In China, there is evidence that the etiological diagnosis of about half of patients with pulmonary infections is unclear (Zhu et al., 2018). The incidence of mixed infections is higher, especially in critically ill or immunocompromised patients. LRTIs are caused by various pathogens, including bacteria, viruses, and other atypical pathogens (Duan et al., 2020). Atypical pathogens such as *Chlamydia psittaci*, *Mycoplasma pneumoniae*, *Legionella pneumophila*, which do not have typical clinical signs or symptoms, are usually impossible to detect using traditional microbial methods (Arnold et al., 2016). For a long time, the diagnosis of LRTIs has mainly relied on traditional microbial methods, antigen and/or antibody immunological methods, and polymerase chain reaction (PCR) detection. However, the traditional pathogen detection capability is limited, it is a time-consuming process, and it has a low detection rate. On the other hand, the antigen and/or antibody immunological method and the PCR technique for pathogen detection must be based on the gene sequences of known pathogens, which means that unknown pathogens cannot be identified. Antimicrobial treatments are mostly empirical, and the delivery of targeted treatments is difficult.

Currently, metagenomic next-generation sequencing (mNGS), an unbiased and culture-independent method, is a high-throughput sequencing technology for the analysis of nucleic acids in samples to detect and identify microbial DNA and/or RNA. mNGS has shown good performance in clinical applications for LRTIs, improving the diagnosis of pulmonary co-infections, shortening the time of detection, and identifying novel and rare pathogens (Leo et al., 2017; Miao et al., 2018; Chiu and Miller, 2019). The utilization of bronchoalveolar lavage fluid (BALF) in mNGS could directly affect the treatment outcomes of patients, including reducing the duration of both antibiotic use and mechanical ventilation (Gaston et al., 2022; Liang et al., 2022). Generally, mNGS is performed by third-party commercial laboratories, and only a few hospitals have attempted to perform the entire process of conducting the mNGS and reporting the results in their own laboratories, i.e., “in-house mNGS.” The results of mNGS may be influenced by library preparations and the use of various sequencing platforms and different bioinformatics analysis methods (Park et al., 2021). In this study, we aimed to evaluate the pathogenic diagnostic value and the potential clinical implications of mNGS based on BALF in a commercial and an in-house laboratory.

## Materials and methods

### Patients and study design

This retrospective study was conducted at Taizhou Hospital of Zhejiang Province from January 2019 to December 2021. Patients with clinical suspicion of LRTIs who underwent BALF mNGS during the study period were enrolled. Patients who met criteria 1–3 and at least one of criteria 4–6 were included, detailed as follows: 1) ≥18 years old; 2) lung imaging showing a new or progressive infiltrate, consolidation, or ground-glass opacity; 3) could tolerate bronchoscopy; 4) exacerbation of respiratory symptoms; 5) with or without fever, cough, expectoration, shortness of breath, and dyspnea; and 6) showing signs of pulmonary consolidation or moist rales (Metlay et al., 2019). Conventional microbiological detection methods and mNGS were performed simultaneously. A total of 306 inpatients were included in this study. According to the different platforms, the participants were divided into two groups: the commercial group (*n* = 150) and the in-house group (*n* = 156). The clinical information of the two groups is shown in **Table 1**. According to the clinical characteristics, underlying diseases, and laboratory results, the patients in the two groups were matched. This study was approved by the Institutional Medical Ethics Committee of Taizhou Hospital of Zhejiang Province.

**Table 1:** Clinical characteristics and laboratory findings of patients.

	Commercial group (*n* = 150)	In-house group (*n* = 156)	*p*-value
Age (years)	62.1 ± 15.3	60.6 ± 14.5	0.393
Male gender, *n* (%)	102 (68.0)	102 (65.4)	0.628
Smoking history	44 (29.3)	38 (24.4)	0.326
Comorbidity, *n* (%)			
Hypertension	53 (35.3)	44 (28.2)	0.180
Diabetes mellitus	33 (22.0)	44 (28.2)	0.211
Respiratory diseases	19 (12.7)	26 (16.7)	0.323
Cardiovascular diseases	48 (32.0)	35 (22.4)	0.060
Digestive system diseases	20 (13.3)	18 (11.5)	0.634
Renal disease	5 (3.3)	13 (8.3)	0.063
Autoimmune diseases	2 (1.3)	6 (3.8)	0.283
Hematological diseases	7 (4.7)	6 (3.8)	0.722
Malignant tumor	27 (18.0)	29 (18.6)	0.894
Laboratory findings			
WBC (10^9^/L)	9.0 (6.0–13.8)	8.0 (6.0–11.0)	0.092
NEU%	82.5 (74–90)	80 (70–89)	0.077
Hb (g/L)	118 (99.5–133.5)	117 (101.8–131)	0.830
PLT (10^9^/L)	213 (146.3–277.5)	222 (156–305.3)	0.135

WBC, white blood count; NEU, neutrophil; Hb, hemoglobin; PLT, platelet.

The conventional methods included as follows: 1) bacterial and fungal cultures; 2) Gram stain, Ziehl–Neelsen stain, weak acid fast stain, and Grocott-methenamine stain were used to identify bacteria, *Mycobacterium tuberculosis* complex (MTBC), *Nocardia*, and *Pneumocystis jirovecii*; 3) other fungi using smear microscopy; 4) PCR and serum antibodies were used for respiratory viruses, cytomegalovirus (CMV) , *M. pneumoniae*, *Chlamydia pneumoniae* and *L. pneumophila*; 5) GeneXpert (Cepheid, Sunnyvale, CA, USA), the DNA microarray method, and the T-SPOT assay were performed in patients with suspicion of *M. tuberculosis*; 6) 1,3-β-D-glucan, galactomannan antigen and cryptococcus antigen test were conducted to identify fungi.

### Sample processing and nucleic acid extraction

BALF was collected from patients according to standard operating procedures and the samples were stored at −20°C.

In the commercial group, DNA was extracted using a Pathogen DNA Kit (JIEYI Biotech, Hangzhou, China) according to the manufacturer’s instructions. Approximately, 600 μl of treated specimens was mixed with glass beads of 0.1–0.2 mm diameter. The tubes were heated at 99°C for 10 min before DNA extraction. Human DNA was removed with Benzonase® (Qiagen, Hilden, Germany) and Tween-20 (Sigma, St. Louis, MO, USA). The differential lysis method was used to remove the host DNA. Subsequently, microbial cells were obtained by enzymatic digestion, followed by wall breaking and nucleic acid extraction (Shi et al., 2020).

In the in-house group, 1.5-ml microcentrifuge tubes with 0.6 ml sample and 250 μl 0.5-mm glass beads were attached to a horizontal platform on a vortex mixer and vigorously agitated at 2,800–3,200 rpm for 30 min. Afterward, 7.2 μl lyticase was added for the wall-breaking reaction. DNA was extracted using the TIANamp Micro DNA Kit (DP316; TIANGEN Biotech, Beijing, China) according to the manufacturer’s recommendation. The extracted DNA specimens were used for the construction of a DNA library (Long et al., 2016).

### Library construction and sequencing

In the commercial group, 1 μg of genomic DNA was taken and interrupted by ultrasound using a Covaris instrument. DNA needs to be fragmented to obtain 200- to 400-bp inserts, followed by terminal repair and A-tailing reactions; finally, PCR amplification of the ligated products was performed (Shi et al., 2020). The sequencing library was prepared using the DNA Library Prep Kit (JIEYI Biotech, Hangzhou, China) according to the manufacturer’s instructions. The Illumina NextSeq CN500 platform was used for sequencing. For each run, we used an environment control sample and different ID spike variants to monitor the contamination of exogenous microorganisms and samples.

In the in-house group, the construction of the DNA library included DNA fragmentation, end repair, adapter ligation, and PCR amplification. Agilent 2100 was used for quality control of the DNA libraries to ensure that the size of the fragments reached up to 300 bp. Quality qualified libraries were pooled and DNA nanoball (DNB) was created and sequenced using the MGISEQ-2000 platform (Liu et al., 2022). Optical signals were collected using a high-resolution imaging system and then converted into digital information, which was then decoded into DNA sequence information. For quality control and to minimize contamination, the positive and negative controls were prepared in parallel and sequenced simultaneously in each batch.

### Bioinformatics analyses

For the commercial group, in order to generate high-quality data, the raw data were subjected to the removal of low-quality reads, adapter contamination, and duplicate reads, as well as the reads shorter than 35 bp, and trimmed using SOAPnuke software. Human host sequences were filtered and excluded by mapping to the human reference genome (hg19) using Bowtie2 (van Boheemen et al., 2020). The remaining data were aligned to a microbial genome database. The RefSeq database contains 3,716 bacterial genomes or scaffolds, 5,272 whole-genome sequences of viral taxa, 247 whole-genome sequences of fungal taxa, and 73 whole-genome sequences of parasites.

For the in-house group, high-quality sequencing data were generated by removing low-quality reads, followed by the identification of human host sequences, which were excluded by mapping to the human reference genome including hg38 and the Yanhuang genome sequence using the Burrows–Wheeler Alignment software (Diao et al., 2022). After the removal of low-complexity reads, the remaining data were classified by aligning to the Pathogens Metagenomics Database (PMDB), which includes bacteria, fungi, viruses, and parasites. The classification reference databases were downloaded from the NCBI database. Thereafter, each sequence was aligned by taxonomic classification for microbial identification. The RefSeq database contains 6,350 bacterial genomes or scaffolds, 4,945 whole-genome sequences of viral taxa, 1,064 whole-genome sequences of fungal taxa, and 234 whole-genome sequences of parasites.

### Criteria for positive mNGS results

At present, there is no uniform standard for the interpretation of mNGS results. The criteria for mNGS positivity have been established according to the literature (Fang et al., 2020; Jin et al., 2022), as follows: 1) >30% relative abundance of bacteria (excluding *M. tuberculosis*) and fungi at the genus level; 2) one or more unique reads of *M. tuberculosis* either at the species or the genus level; 3) a stringent map read number (SMRN) of ≥3 for viruses; and 4) positive culture and/or histopathological examination and at least 50 unique reads from a single species of bacteria or fungi. The final etiology confirmation of all cases was decided by a group of clinical microbiologists and physicians.

### Statistical analyses

Student’s *t*-test (for normally distributed variables) and the Mann–Whitney *U* test (for variables with non-normal distribution) were used to compare the quantitative data between the two groups; categorical variables were compared using the chi-square test. A *p* < 0.05 was considered significant. Data analyses were performed using SPSS 23.0 (SPSS Inc., Chicago, IL, USA), and figures were constructed using GraphPad Prism 7 software.

## Results

**Figure 1:**
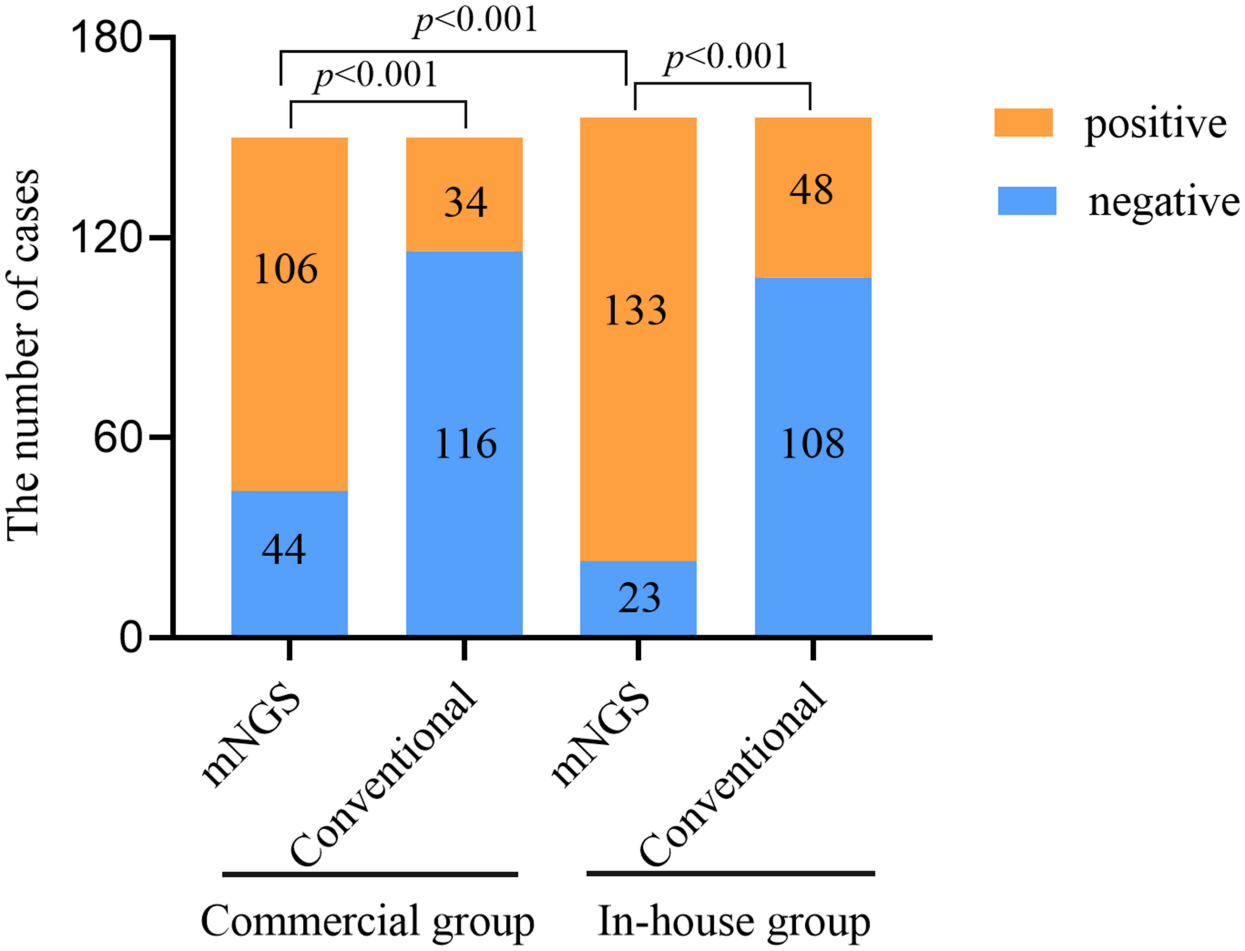
Comparison of the positivity rates between metagenomic next-generation sequencing (mNGS) and conventional tests.

The positivity rates of BALF mNGS and conventional tests for the two groups are shown in **Figure 1**. In the commercial group, the positive results of mNGS and conventional tests were 70.67% and 22.67%, respectively, with the positivity rate of mNGS being significantly higher than that of conventional methods (*p* < 0.001). In the in-house group, the positivity rate of mNGS was higher than that of conventional methods (85.26% *vs*. 30.77%, *p* < 0.001). Similarly, the positivity rate of mNGS in the in-house group was significantly higher than that in the commercial group (*p* < 0.001).

**Figure 2:**
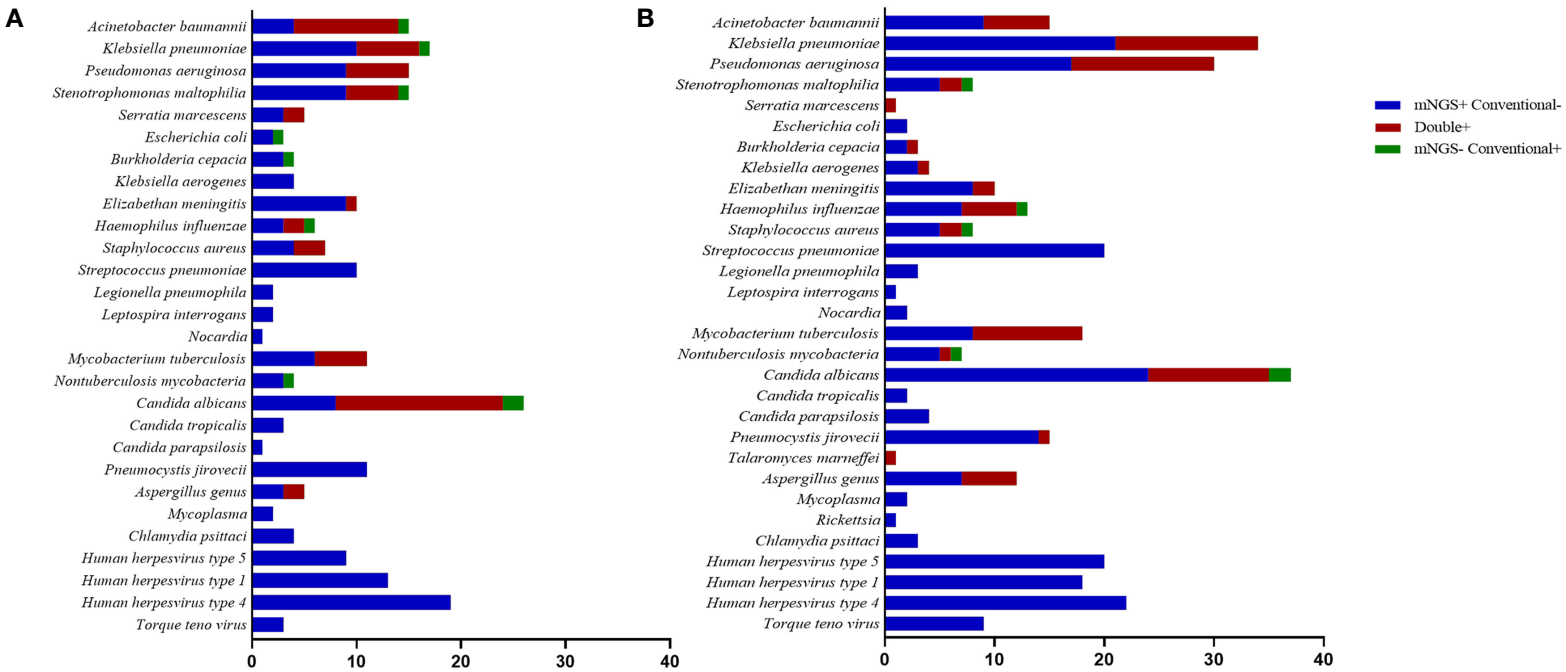
Species distribution of bacteria, fungi, and other pathogens detected using metagenomic next-generation sequencing (mNGS) and conventional tests. **(A)** Commercial group. **(B)** In-house group.

**Figure 2** shows the pathogen spectrum detected using mNGS and conventional tests. In the results, *Klebsiella pneumoniae* (*n* = 16) was the most commonly detected bacteria by mNGS in the commercial group, followed by *Pseudomonas aeruginosa* (*n* = 15) and *Stenotrophomonas maltophilia* (*n* = 14). We also detected 11 cases of *M. tuberculosis* using mNGS. The most common fungal species detected were *Candida* (28/150, 18.7%), *P. jirovecii* (11/150, 7.3%), and *Aspergillus* (5/150, 3.3%). *Human herpesvirus types 1* and *4* were the main viruses detected. In the in-house group, *K. pneumoniae* (*n* = 34) was the major bacteria identified using mNGS, followed by *P. aeruginosa* (*n* = 30) and *Streptococcus pneumoniae* (*n* = 20). We also detected 24 cases of *M. tuberculosis* using mNGS. The most prevalent pathogenic fungal species were *Candida* (41/156, 26.3%), *P. jirovecii* (15/156, 9.6%), *Aspergillus* (12/156, 7.7%), and *Penicillium marneffei* (1/156, 0.6%). There were more viruses detected using mNGS in the commercial group than in the in-house group. In the positive conventional tests, the most common pathogens detected were *Acinetobacter baumannii*, *K. pneumoniae*, *P. aeruginosa*, and *Candida*. However, viruses and atypical pathogens could not be identified using traditional methods.

**Figure 3:**
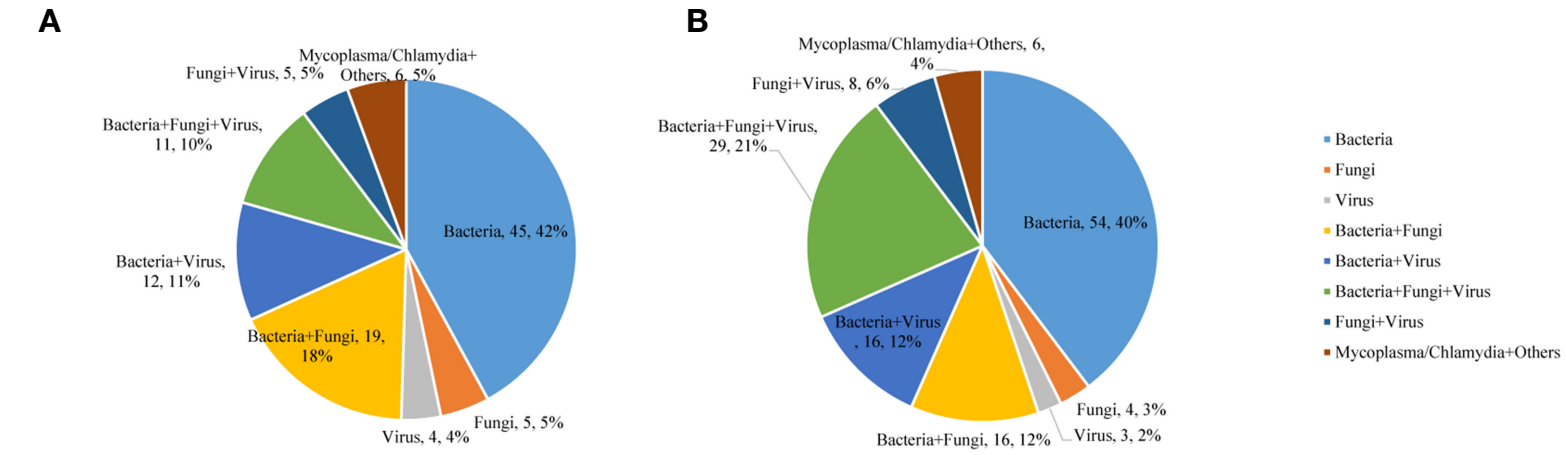
Mixed infections for various pathogens detected using metagenomic next-generation sequencing (mNGS). **(A)** Commercial group. **(B)** In-house group.

**Figure 4:**
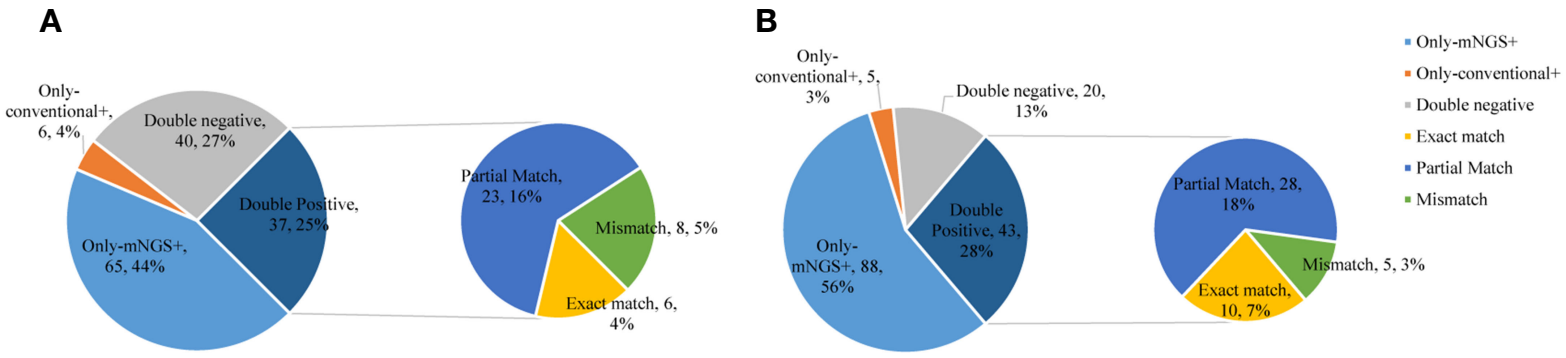
Comparison of the consistency between metagenomic next-generation sequencing (mNGS) and conventional tests. **(A)** Commercial group. **(B)** In-house group.

In the commercial group, the proportion of single pathogens was 51%. The most frequent pathogen was bacteria, accounting for 42%, and the proportion of multiple pathogens was 49%. Multiple pathogens also comprised a higher percentage in the in-house group (55%) (**Figure 3**). In addition, in the commercial group, the mNGS and conventional methods showed consistent results in detection, with both positive for pathogens in 37 cases and negative in 40 cases. Of the 37 cases found positive by both methods, the results of mNGS and conventional methods were completely matched in six cases, partially matched in 23 cases, and completely mismatched in eight cases. Out of 150 cases, a total of 65 (44%) were positive by mNGS, while only 6 (4%) cases were positive by conventional methods. On the other hand, in the in-house group, both the mNGS and conventional methods showed positive pathogen detection in 43 cases and negative in 20 cases. Of the 43 cases detected positive by both methods, the results of mNGS and conventional methods were completely matched in 10 cases, partially matched in 28 cases, and were completely mismatched in five cases. Out of 156 cases, a total of 88 (56%) were positive for pathogens by mNGS, which was higher than that in the commercial group, while only 5 (3%) cases were positive for pathogens by conventional methods (**Figure 4**).

**Figure 5:**
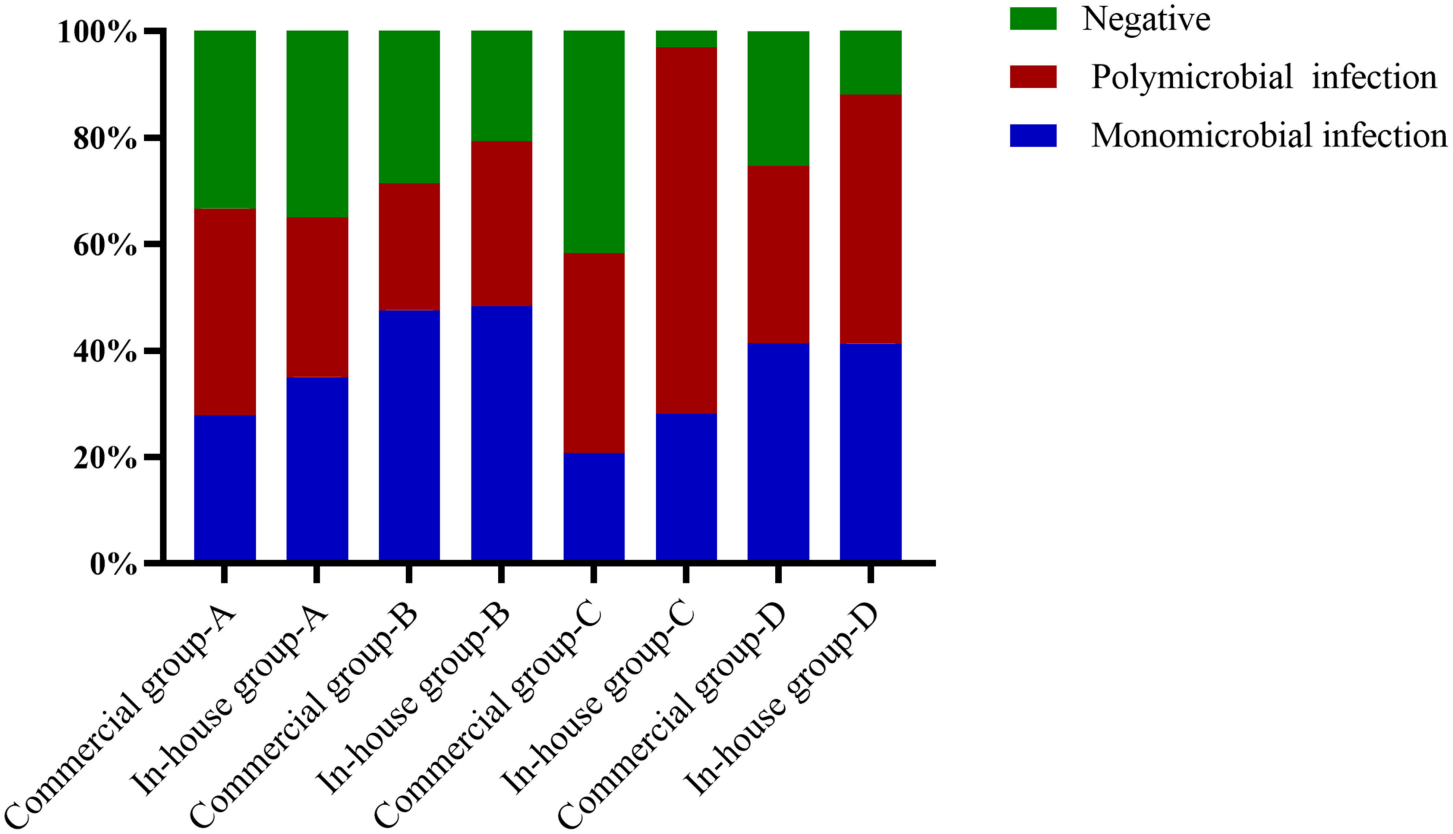
Proportions of polymicrobial and monomicrobial infections and negative cases of pulmonary infection detected using metagenomic next-generation sequencing (mNGS) in patients with different underlying diseases. **(A)** Simple pulmonary infection group. **(B)** Chronic airway infection group. **(C)** Immunocompromised group. **(D)** Others.

The main type of infection in the the simple pulmonary infection and chronic airway inflammation group was a simple bacterial infection, while that in the immunocompromised group was a mixed infection. Except for the simple pulmonary infection group, the positive rate and the detection rate of multiple microorganisms in the in-house group were higher than those in the commercial group (**Figure 5**).

**Figure 6:**
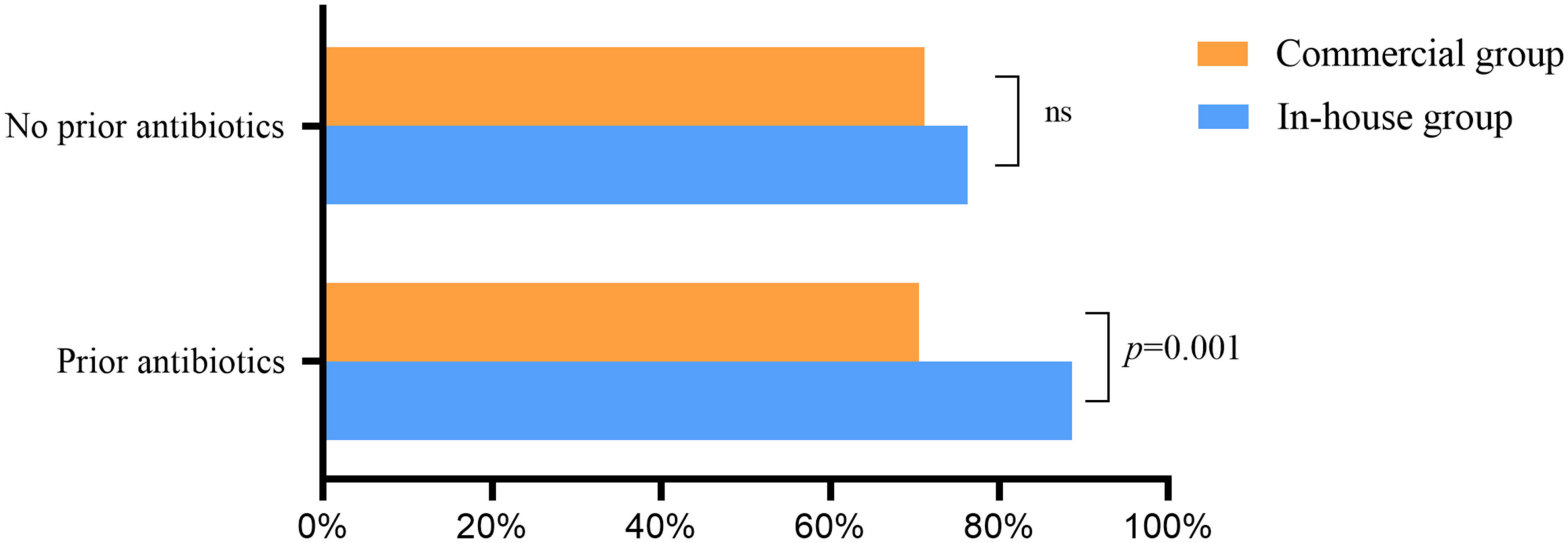
Effect of previous antibiotic exposure on the positivity rate of metagenomic next-generation sequencing (mNGS) ns, no significance.

Without empirical therapy, there was no significant difference in the positive rate of mNGS between the commercial and in-house groups (**Figure 6**). With prior antibiotic exposure, the positive rate of mNGS in the in-house group was higher than that in the commercial group (88.6% *vs*. 70.5%, *p* = 0.001).

**Figure 7:**
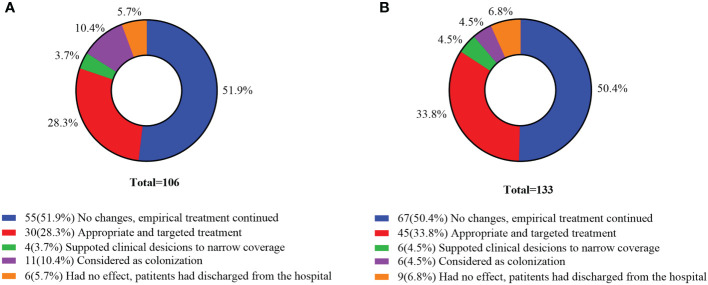
Clinical impact of metagenomic next-generation sequencing (mNGS) positive results. **(A)** Commercial group. **(B)** In-house group.

Of the 106 mNGS positive cases in the commercial group, 55 (51.9%) cases had no changes and empirical therapy continued, 34 (32.0%) cases had their antibiotics adjusted, and 11 cases were considered as colonization. Among the 133 mNGS positive cases in the in-house group, 67 (50.4%) cases had no changes and empirical therapy continued, 45 (33.8%) cases received targeted treatment, and 6 (4.5%) cases supported clinical considerations to narrow coverage. According to the results of mNGS, the proportion of antibiotics adjusted in the in-house group was higher than that in the commercial group (**Figure 7**).

The turnaround time of mNGS in the in-house group was significantly shorter than that in the commercial group. In the chronic airway infection group and others group, the time from mNGS to discharge in the in-house group was significantly shorter than that in the commercial group (**Table 2**).

**Table 2:** Comparison of the mNGS turnaround time and the discharge time of patients.

	Commercial group (*n* = 150)	In-house group (*n* = 156)	*p*-value
mNGS turnaround time (days)	3.05 ± 0.68	1.87 ± 0.73	**<0.001**
Time from mNGS to discharge (days)	10(3-18)	6(2-12)	**0.001**
Simple pulmonary infection group	3(1-10)	1(1-4)	0.151
Chronic airway infection group	10(3-13.5)	4(1-6.5)	**0.006**
Immunocompromised group	10(7-17.8)	10.5(4-13.5)	0.823
Others	12(5-21)	6.5(3-14)	**0.005**
Mortality, n (%)	33(21.2)	31(20.7)	0.917

The bold values indicate statistical significance (p < 0.05).

## Discussion

Some refractory LRTIs are difficult to diagnose and the pathogens are not clear, causing severe challenges to microbiological diagnosis and clinical treatments (Langelier et al., 2018; Wu et al., 2020). A prospective study in Europe showed that the etiology of 40% of LRTIs was still unclear even with the combination of microbial culture, PCR, and serological methods (Ieven et al., 2018). In recent years, mNGS has shown the advantages of being more efficient and accurate for pathogen diagnosis, thus updating the diagnostic strategy for LRTIs (Zhang et al., 2019). We reported a retrospective study on the application of mNGS in the diagnosis of infectious pathogens with BALF in commercial and in-house laboratories. Compared with conventional tests, BALF mNGS showed a significantly higher positive rate and a wider pathogen spectrum, which was consistent with a previous study (Li et al., 2020). The results showed that the positive rate of mNGS in the in-house group (85.26%) was significantly higher than that in the commercial group (70.67%), which may be related to differences in the extraction methods and the sequencing platform.

This study found that the detection rate of mNGS was high for bacteria, but low for fungi and viruses. Among the single pathogens of LRTIs, bacteria played a major role. mNGS showed an advantage in the diagnosis of mixed pulmonary infections (Pan et al., 2019). Wang et al. (2019) showed that the sensitivity of mNGS in the diagnosis of mixed pulmonary infections (97.2% *vs*. 13.9%, *p* < 0.01) was significantly higher than that of routine detection. Zhao et al. (2021) diagnosed 119 patients with a pulmonary fungal infection, with 48 cases (40.3%) being complicated by both pulmonary fungal and bacterial infections. The results of this study showed that the proportion of mixed pathogens in the in-house group was 55%, which was higher than that in the commercial group. This suggests that our in-house platform had obvious advantages in detecting bacterial, fungal, and viral co-infections. This advantage may help clinicians identify pulmonary mixed infections, evaluate patients more comprehensively, and make effective treatment decisions.

LRTIs are caused by hundreds of pathogens, especially those that are rare and difficult to cultivate, such as *Leptospira*, *Legionella*, and *C. psittaci*. Previous studies have mostly recommended serum antibody detection and PCR methods. Molecular and/or serological microbiological investigations are based on targeted microorganisms, which may miss the detection of potential atypical pathogens. In the past, atypical pathogens were thought to be relatively rare. After the application of mNGS, the detection rate of atypical pathogens has increased greatly. In this study, two cases of *Leptospira* spp., two cases of *L. pneumophila*, and four cases of *C. psittaci* were detected in the commercial group, while one case of *Leptospira interrogans*, three cases of *L. pneumophila*, and three cases of *C. psittaci* were detected in the in-house group; most cases were accompanied by infection with other pathogens. Therefore, the in-house platform showed no advantage in the detection of atypical pathogens.

This study found that mNGS identified more fungi and *M. tuberculosis* in the in-house group compared to the commercial group. Furthermore, 11 cases of *P. jirovecii*, five cases of *Aspergillus*, and 11 cases of *M. tuberculosis* were detected in the commercial group. More cases were detected in the in-house group: 15 cases of *P. jirovecii*, 12 cases of *Aspergillus*, one case of *P. marneffei*, and 18 cases of *M. tuberculosis*. The results showed that, in the in-house group, mNGS significantly improved the detection rate of fungi and *M. tuberculosis*. According to previous research (Li et al., 2018; Liu et al., 2021), the combination of mNGS and conventional tests can improve the detection rate of *fungi* and *M. tuberculosis*. It is difficult for common pathogens such as fungi (with a complex cell structure and cell wall), *M. tuberculosis* (with mycotic acid outside the cell wall and a large genome), and intracellular parasitic bacteria to break the wall, resulting in a low nucleic acid sequence abundance that is difficult to detect. The positive rate in the in-house group was improved with improvement in the wall breaking technique.

Due to the different immune functions of the host and nonspecific syndrome, a sensitive and comprehensive method for the detection of viral pathogens is extremely important. In many cases, the presence of a DNA virus, such as the Torque teno virus (TTV), is considered to be related to the low immunity of patients (Maggi et al., 2011; Görzer et al., 2014). A previous study showed that solid organ transplant recipients with viral and bacterial co-infections had poor prognoses (Pan et al., 2022). mNGS can also detect viruses that cannot be detected by conventional methods; seven types of respiratory DNA viruses were identified by mNGS, with the most commonly detected virus being the human herpesvirus. The virus was detected in 28 patients in the commercial group and 55 patients in the in-house group. The detection of viruses requires sufficient sequencing length and depth. These results indicate that the in-house platform had obvious advantages in virus detection.

The positive rate and the detection rate of multiple microorganisms in the in-house group were high. Firstly, this was related to the different DNA extraction methods. Different DNA extraction methods could lead to different DNA yields. An increased yield of pathogen DNA would also likely improve performance (Govender et al., 2021). The differential lysis method was used to remove the host DNA in the commercial group, which could have removed part of the pathogen DNA in cells, and showed low sensitivity for the detection of viruses and parasites (eukaryotes) (Miller and Chiu, 2021). Secondly, there were differences in the read length and sequencing data between the Illumina NextSeq CN500 and MGISEQ-2000 platforms. The Illumina NextSeq series sequence platform uses a bridge amplification strategy, while the MGISEQ platform clones single-stranded circular DNA using rolling circle amplification to produce DNBs (Zhu et al., 2021; Diao et al., 2022). Thirdly, different laboratories vary in terms of quality control, coupled with the presence of microbial contaminants in the reagents used for processing or in the laboratory environment, which may complicate the analysis and interpretation of the results. At present, there is a lack of uniform standards, and each laboratory has a set of criteria for the interpretation of the mNGS results. In addition to the above considerations, in this study, the interpretation of the results was “normalized,” each sample had the same criteria and was combined with clinical symptoms, the results of the conventional methods, and laboratory indicators.

Previous use of broad-spectrum antibiotics can lead to “false-negative” results for microorganism culture. However, in this study, previous antibiotic exposure had little effect on the mNGS results, which was consistent with Miao et al. (2018). mNGS can identify pathogens and guide clinicians in adjusting the dosage of antibiotics. Our results showed that the percentages of antibiotics modified according to the results of mNGS in the commercial and in-house groups were 32.0% and 38.3%, respectively. The time from mNGS to discharge in the in-house group was significantly shorter than that in the commercial group. Xie et al. (2019) showed that the 28- and 90-day mortality rate of the mNGS group was significantly lower than that of the conventional detection group. Chen et al. (2020) also reported that in-house mNGS identified more causative organisms and a 2-day turnaround time, which had higher clinical utility. The turnaround time of mNGS in the commercial group was 3.05 ± 0.68 days, while that in the in-house group was 1.87 ± 0.73 days, which was noticeably shorter. The rapid turnaround time of in-house mNGS is more useful for providing appropriate treatments, for early discharge of patients, and for supporting clinical decisions.

This study had some limitations. Firstly, this is a single-center retrospective study with a relatively small sample size. Secondly, only DNA sequencing was carried out, so that RNA viruses were not detected. Finally, the respiratory microbiome data were not obtained from a healthy population as a baseline microbial community; therefore, it was difficult to characterize the respiratory microbiome of patients and to generate an optimal threshold for pathogen identification.

## Conclusion

The mNGS technique identified more infectious pathogens than conventional methods. In addition, the in-house platform had potential advantages: the positive rate was higher and the pathogen spectrum wider than those in the commercial group, and the turnaround time was also shorter, which accelerates clinical decision-making and shortens hospitalization. This study highlighted the performance of mNGS in the in-house laboratory as a powerful complement to conventional methods in clinical applications due to its enhanced spectrum of microbiological diagnosis and high clinical utility.

## Data availability statement

The datasets presented in this study can be found in online repositories. The names of the repository/repositories and accession number(s) can be found below: CNSA, CNP0003250.

## Ethics statement

The studies involving human participants were reviewed and approved by the Institutional Medical Ethics Committee of Taizhou Hospital of Zhejiang Province. Written informed consent was obtained from each patient before performing bronchoscopy, and identifying information was removed.

## Author contributions

SL and SY had roles in the study design, data analysis, literature search, and writing of the manuscript. BS and T-HT had roles in guiding research and clinical management. JQin, PZ, JQian, MP, YC and QS had roles in data collection and interpretation. All authors contributed to the article and approved the submitted version.
